# Supplementary material for: Hot spot prediction in protein-protein interactions by an ensemble system
Source: BMC Syst Biol. 2018 Dec 31;12(Suppl 9):132. doi: 10.1186/s12918-018-0665-8 (PMC6311905; doi:10.1186/s12918-018-0665-8)
Supplement: Supplementary file 2 — All relevant MATLAB code. A simple Matlab implement of our predictor. (ZIP 19,742 kb) [file 12918_2018_665_MOESM2_ESM.zip › Additional file 2/Read ASA/ASA website/dbMPIKT/4QTIU.html]

 NetSurfP - Protein Surface Accessibility and Secondary Structure Predictions

  

|  |  |  |
| --- | --- | --- |
|  |  | NetSurfP - Protein Surface Accessibility and Secondary Structure Predictions  Technical University of Denmark |

  


---

```
# For publication of results, please cite:
# A generic method for assignment of reliability scores applied to solvent accessibility predictions.
# Bent Petersen, Thomas Nordahl Petersen, Pernille Andersen, Morten Nielsen and Claus Lundegaard
# BMC Structural Biology 2009, 9:51 doi:10.1186/1472-6807-9-51
#
# Column 1: Class assignment - B for buried or E for Exposed - Threshold: 25% exposure, but not based on RSA
# Column 2: Amino acid
# Column 3: Sequence name
# Column 4: Amino acid number
# Column 5: Relative Surface Accessibility - RSA
# Column 6: Absolute Surface Accessibility
# Column 7: Z-fit score for RSA prediction
# Column 8: Probability for Alpha-Helix
# Column 9: Probability for Beta-strand
# Column 10: Probability for Coil
E L  4QTIU                  1    0.582 106.619  -1.689   0.003   0.003   0.994
E R  4QTIU                  2    0.528 120.912   0.927   0.005   0.336   0.660
B C  4QTIU                  3    0.183  25.651  -2.928   0.004   0.514   0.481
E M  4QTIU                  4    0.327  65.433   0.153   0.003   0.718   0.279
E Q  4QTIU                  5    0.520  92.818   0.119   0.021   0.756   0.223
B C  4QTIU                  6    0.103  14.503  -0.833   0.021   0.451   0.528
E K  4QTIU                  7    0.642 132.142  -0.572   0.021   0.279   0.699
E T  4QTIU                  8    0.512  70.987  -0.842   0.004   0.085   0.910
E N  4QTIU                  9    0.552  80.784  -1.004   0.018   0.047   0.935
E G  4QTIU                 10    0.482  37.957  -0.990   0.018   0.047   0.935
E D  4QTIU                 11    0.562  81.042   0.692   0.004   0.138   0.858
B C  4QTIU                 12    0.075  10.586  -0.119   0.022   0.552   0.426
E R  4QTIU                 13    0.387  88.692   1.394   0.021   0.756   0.223
B V  4QTIU                 14    0.235  36.181   0.224   0.018   0.846   0.136
E E  4QTIU                 15    0.449  78.353   0.322   0.018   0.846   0.136
E E  4QTIU                 16    0.555  96.976  -0.114   0.021   0.756   0.223
B C  4QTIU                 17    0.069   9.744  -0.278   0.022   0.359   0.619
E A  4QTIU                 18    0.408  44.962  -0.084   0.056   0.142   0.802
E L  4QTIU                 19    0.321  58.775  -0.629   0.053   0.043   0.903
E G  4QTIU                 20    0.389  30.646  -1.387   0.115   0.016   0.868
E Q  4QTIU                 21    0.390  69.618   0.471   0.257   0.016   0.727
E D  4QTIU                 22    0.463  66.675  -0.823   0.354   0.048   0.598
B L  4QTIU                 23    0.083  15.142   0.421   0.354   0.048   0.598
B C  4QTIU                 24    0.050   7.048  -0.182   0.502   0.102   0.396
E R  4QTIU                 25    0.298  68.334   0.485   0.538   0.173   0.289
B T  4QTIU                 26    0.213  29.557  -0.407   0.453   0.248   0.299
B T  4QTIU                 27    0.142  19.626   0.827   0.478   0.309   0.214
B I  4QTIU                 28    0.089  16.428  -0.540   0.373   0.401   0.226
B V  4QTIU                 29    0.123  18.874   0.118   0.268   0.505   0.227
E R  4QTIU                 30    0.357  81.822   0.180   0.268   0.505   0.227
E L  4QTIU                 31    0.267  48.961   0.595   0.159   0.516   0.325
B W  4QTIU                 32    0.145  34.824   0.249   0.159   0.516   0.325
E E  4QTIU                 33    0.597 104.261   0.211   0.066   0.296   0.638
E E  4QTIU                 34    0.678 118.394  -0.381   0.052   0.084   0.864
E G  4QTIU                 35    0.422  33.243  -1.341   0.053   0.043   0.903
E E  4QTIU                 36    0.621 108.471  -0.031   0.056   0.142   0.802
E E  4QTIU                 37    0.468  81.795   0.470   0.074   0.484   0.442
B L  4QTIU                 38    0.202  37.059   0.484   0.079   0.592   0.329
E E  4QTIU                 39    0.438  76.606   0.071   0.087   0.683   0.230
B L  4QTIU                 40    0.240  43.999   0.521   0.072   0.855   0.073
B V  4QTIU                 41    0.197  30.340   0.183   0.018   0.846   0.136
E E  4QTIU                 42    0.475  82.965  -0.425   0.085   0.775   0.140
E K  4QTIU                 43    0.435  89.397   0.279   0.079   0.592   0.329
E S  4QTIU                 44    0.370  43.399  -1.597   0.066   0.296   0.638
B C  4QTIU                 45    0.134  18.884  -0.356   0.064   0.216   0.721
E T  4QTIU                 46    0.435  60.279  -0.849   0.021   0.279   0.699
E H  4QTIU                 47    0.307  55.789   0.017   0.019   0.141   0.840
E S  4QTIU                 48    0.571  66.945  -0.570   0.018   0.088   0.893
E E  4QTIU                 49    0.711 124.177  -0.386   0.053   0.043   0.903
E K  4QTIU                 50    0.518 106.491  -0.205   0.052   0.084   0.864
E T  4QTIU                 51    0.483  66.978  -0.926   0.052   0.084   0.864
E N  4QTIU                 52    0.479  70.067  -1.274   0.052   0.084   0.864
E R  4QTIU                 53    0.361  82.692  -0.486   0.056   0.142   0.802
E T  4QTIU                 54    0.351  48.628  -0.881   0.066   0.296   0.638
B L  4QTIU                 55    0.180  32.995  -0.167   0.074   0.484   0.442
B S  4QTIU                 56    0.269  31.503  -0.905   0.079   0.592   0.329
B Y  4QTIU                 57    0.221  47.249  -0.581   0.159   0.516   0.325
E R  4QTIU                 58    0.319  72.959  -0.652   0.159   0.516   0.325
B T  4QTIU                 59    0.277  38.392  -0.754   0.135   0.317   0.548
B G  4QTIU                 60    0.272  21.399  -1.392   0.135   0.317   0.548
E L  4QTIU                 61    0.336  61.540  -1.262   0.148   0.418   0.435
E K  4QTIU                 62    0.369  75.903   0.069   0.087   0.683   0.230
B I  4QTIU                 63    0.149  27.639  -0.241   0.085   0.775   0.140
B T  4QTIU                 64    0.178  24.744  -0.087   0.087   0.683   0.230
E S  4QTIU                 65    0.306  35.840  -0.304   0.169   0.612   0.219
B L  4QTIU                 66    0.138  25.286   0.233   0.148   0.418   0.435
E T  4QTIU                 67    0.383  53.178  -0.189   0.231   0.330   0.439
E E  4QTIU                 68    0.381  66.491  -0.513   0.231   0.330   0.439
B V  4QTIU                 69    0.224  34.460  -0.479   0.231   0.330   0.439
B V  4QTIU                 70    0.140  21.441  -0.074   0.252   0.423   0.325
B C  4QTIU                 71    0.120  16.806  -0.282   0.252   0.423   0.325
E G  4QTIU                 72    0.345  27.159  -1.444   0.216   0.235   0.548
B L  4QTIU                 73    0.239  43.852  -1.057   0.118   0.150   0.732
E D  4QTIU                 74    0.386  55.579  -1.298   0.113   0.087   0.800
B L  4QTIU                 75    0.159  29.058  -0.785   0.113   0.087   0.800
B C  4QTIU                 76    0.129  18.168  -1.215   0.053   0.043   0.903
E N  4QTIU                 77    0.425  62.191  -1.442   0.053   0.043   0.903
E Q  4QTIU                 78    0.503  89.782  -0.288   0.058   0.017   0.925
E G  4QTIU                 79    0.550  43.285  -1.571   0.018   0.019   0.964
E N  4QTIU                 80    0.578  84.663  -1.616   0.018   0.019   0.964
E S  4QTIU                 81    0.534  62.585  -1.988   0.018   0.019   0.964
E G  4QTIU                 82    0.382  30.063  -1.809   0.018   0.019   0.964
E R  4QTIU                 83    0.522 119.652  -1.102   0.018   0.047   0.935
E A  4QTIU                 84    0.550  60.588  -2.335   0.018   0.047   0.935
E V  4QTIU                 85    0.410  63.078  -0.304   0.018   0.088   0.893
E T  4QTIU                 86    0.483  66.937  -0.812   0.019   0.141   0.840
B Y  4QTIU                 87    0.113  24.063   0.045   0.018   0.088   0.893
E S  4QTIU                 88    0.464  54.369  -0.987   0.018   0.047   0.935
E R  4QTIU                 89    0.491 112.439  -1.176   0.018   0.047   0.935
E S  4QTIU                 90    0.321  37.633  -1.726   0.018   0.088   0.893
E R  4QTIU                 91    0.337  77.196   0.215   0.021   0.279   0.699
B Y  4QTIU                 92    0.235  50.155  -0.440   0.021   0.756   0.223
B L  4QTIU                 93    0.068  12.451   0.578   0.018   0.846   0.136
B E  4QTIU                 94    0.273  47.763   0.246   0.018   0.846   0.136
B C  4QTIU                 95    0.036   5.082  -0.780   0.018   0.846   0.136
B I  4QTIU                 96    0.267  49.395  -0.209   0.021   0.756   0.223
E S  4QTIU                 97    0.400  46.892  -0.368   0.022   0.552   0.426
B C  4QTIU                 98    0.086  12.046  -0.624   0.020   0.205   0.775
E G  4QTIU                 99    0.505  39.767  -0.394   0.018   0.047   0.935
E S  4QTIU                100    0.553  64.812  -0.650   0.018   0.047   0.935
E S  4QTIU                101    0.516  60.522  -1.051   0.018   0.047   0.935
E D  4QTIU                102    0.593  85.480  -1.696   0.053   0.043   0.903
E M  4QTIU                103    0.404  80.800  -1.206   0.053   0.043   0.903
E S  4QTIU                104    0.369  43.223  -1.337   0.053   0.043   0.903
B C  4QTIU                105    0.134  18.771  -1.010   0.113   0.087   0.800
E E  4QTIU                106    0.522  91.106  -0.355   0.113   0.043   0.844
E R  4QTIU                107    0.559 128.011  -0.304   0.113   0.043   0.844
E G  4QTIU                108    0.526  41.428  -1.179   0.053   0.043   0.903
E R  4QTIU                109    0.434  99.363   0.175   0.052   0.084   0.864
E H  4QTIU                110    0.566 102.955  -1.175   0.022   0.359   0.619
E Q  4QTIU                111    0.392  70.083   0.368   0.023   0.655   0.322
E S  4QTIU                112    0.519  60.874  -0.232   0.021   0.756   0.223
B L  4QTIU                113    0.079  14.483   0.144   0.021   0.756   0.223
E Q  4QTIU                114    0.563 100.534   0.418   0.004   0.616   0.381
B C  4QTIU                115    0.046   6.416  -1.024   0.004   0.197   0.799
E R  4QTIU                116    0.493 112.783  -0.328   0.005   0.045   0.951
E S  4QTIU                117    0.458  53.666  -0.635   0.018   0.019   0.964
E P  4QTIU                118    0.470  66.721  -0.796   0.018   0.019   0.964
E E  4QTIU                119    0.486  84.922  -0.329   0.058   0.017   0.925
E E  4QTIU                120    0.602 105.187   0.647   0.118   0.150   0.732
E Q  4QTIU                121    0.296  52.955   1.154   0.148   0.418   0.435
B C  4QTIU                122    0.034   4.802  -0.067   0.268   0.505   0.227
B L  4QTIU                123    0.176  32.299   1.021   0.273   0.587   0.140
E D  4QTIU                124    0.315  45.392  -0.308   0.391   0.466   0.143
B V  4QTIU                125    0.111  17.091   0.625   0.373   0.401   0.226
B V  4QTIU                126    0.086  13.187   0.233   0.268   0.505   0.227
B T  4QTIU                127    0.236  32.705  -0.511   0.273   0.587   0.140
B H  4QTIU                128    0.239  43.383  -0.069   0.273   0.587   0.140
B W  4QTIU                129    0.212  50.986   0.207   0.268   0.505   0.227
E I  4QTIU                130    0.323  59.700  -0.671   0.373   0.401   0.226
E Q  4QTIU                131    0.399  71.226  -1.262   0.321   0.252   0.427
E E  4QTIU                132    0.338  58.961  -0.420   0.307   0.165   0.527
E G  4QTIU                133    0.323  25.404  -1.319   0.118   0.150   0.732
E E  4QTIU                134    0.537  93.884  -1.829   0.052   0.084   0.864
E E  4QTIU                135    0.610 106.532  -1.440   0.056   0.142   0.802
E G  4QTIU                136    0.403  31.732  -1.150   0.064   0.216   0.721
E R  4QTIU                137    0.331  75.913  -0.960   0.056   0.142   0.802
E P  4QTIU                138    0.297  42.144  -1.024   0.052   0.084   0.864
E K  4QTIU                139    0.525 108.095  -0.755   0.052   0.084   0.864
E D  4QTIU                140    0.550  79.241  -1.455   0.052   0.084   0.864
E D  4QTIU                141    0.495  71.344  -1.159   0.056   0.142   0.802
E R  4QTIU                142    0.412  94.302  -0.030   0.066   0.296   0.638
E H  4QTIU                143    0.309  56.298   0.019   0.022   0.552   0.426
B L  4QTIU                144    0.236  43.248   0.138   0.022   0.552   0.426
E R  4QTIU                145    0.374  85.577   0.056   0.022   0.359   0.619
B G  4QTIU                146    0.279  21.926  -1.643   0.021   0.279   0.699
B C  4QTIU                147    0.097  13.563  -0.638   0.020   0.205   0.775
B G  4QTIU                148    0.220  17.322  -2.166   0.020   0.205   0.775
E Y  4QTIU                149    0.378  80.779  -1.155   0.019   0.141   0.840
B L  4QTIU                150    0.251  45.995  -1.608   0.018   0.047   0.935
E P  4QTIU                151    0.639  90.646  -2.123   0.018   0.019   0.964
E G  4QTIU                152    0.432  34.006  -2.290   0.005   0.015   0.979
B C  4QTIU                153    0.091  12.804  -0.657   0.018   0.019   0.964
E P  4QTIU                154    0.373  52.915  -1.613   0.018   0.019   0.964
B G  4QTIU                155    0.301  23.665  -2.301   0.018   0.019   0.964
E S  4QTIU                156    0.398  46.657  -1.668   0.018   0.047   0.935
E N  4QTIU                157    0.356  52.104  -2.742   0.053   0.043   0.903
E G  4QTIU                158    0.376  29.591  -2.371   0.053   0.043   0.903
B F  4QTIU                159    0.217  43.552  -2.982   0.053   0.043   0.903
B H  4QTIU                160    0.303  55.152  -2.042   0.053   0.043   0.903
E N  4QTIU                161    0.358  52.440  -2.289   0.058   0.017   0.925
B N  4QTIU                162    0.199  29.148  -0.980   0.181   0.016   0.803
E D  4QTIU                163    0.300  43.172  -0.282   0.268   0.043   0.689
B T  4QTIU                164    0.186  25.784  -0.477   0.307   0.165   0.527
B F  4QTIU                165    0.103  20.592  -0.271   0.321   0.252   0.427
B H  4QTIU                166    0.231  42.073  -0.141   0.352   0.332   0.316
B F  4QTIU                167    0.212  42.508  -0.824   0.352   0.332   0.316
B L  4QTIU                168    0.107  19.592  -0.298   0.231   0.330   0.439
E K  4QTIU                169    0.357  73.497   0.655   0.231   0.330   0.439
B C  4QTIU                170    0.171  23.994  -0.134   0.231   0.330   0.439
B C  4QTIU                171    0.079  11.035  -0.047   0.216   0.235   0.548
E N  4QTIU                172    0.442  64.709  -0.283   0.191   0.086   0.723
E T  4QTIU                173    0.446  61.874  -0.157   0.278   0.093   0.628
E T  4QTIU                174    0.453  62.859  -1.022   0.278   0.093   0.628
E K  4QTIU                175    0.367  75.574   0.477   0.184   0.043   0.773
B C  4QTIU                176    0.113  15.921  -1.740   0.191   0.086   0.723
E N  4QTIU                177    0.451  66.056  -1.025   0.113   0.043   0.844
E E  4QTIU                178    0.609 106.375  -1.937   0.053   0.043   0.903
E G  4QTIU                179    0.552  43.466  -0.846   0.053   0.043   0.903
E P  4QTIU                180    0.424  60.194  -0.737   0.053   0.043   0.903
B I  4QTIU                181    0.264  48.877  -0.719   0.053   0.043   0.903
B L  4QTIU                182    0.179  32.702  -0.817   0.018   0.047   0.935
E E  4QTIU                183    0.530  92.661   0.622   0.018   0.047   0.935
B L  4QTIU                184    0.269  49.309  -1.722   0.018   0.088   0.893
E E  4QTIU                185    0.560  97.780  -0.540   0.018   0.047   0.935
E N  4QTIU                186    0.692 101.338  -1.114   0.018   0.047   0.935
B L  4QTIU                187    0.261  47.844  -0.459   0.018   0.019   0.964
E P  4QTIU                188    0.447  63.373  -0.530   0.018   0.019   0.964
E Q  4QTIU                189    0.527  94.069  -1.321   0.058   0.017   0.925
E N  4QTIU                190    0.509  74.488  -1.276   0.058   0.017   0.925
E G  4QTIU                191    0.390  30.717  -1.699   0.113   0.043   0.844
E R  4QTIU                192    0.383  87.730  -0.854   0.056   0.142   0.802
E Q  4QTIU                193    0.429  76.673   0.237   0.069   0.386   0.545
B C  4QTIU                194    0.101  14.152  -0.268   0.079   0.592   0.329
B Y  4QTIU                195    0.188  40.197   0.272   0.023   0.655   0.322
B S  4QTIU                196    0.244  28.538  -0.994   0.079   0.592   0.329
B C  4QTIU                197    0.115  16.132  -0.914   0.069   0.386   0.545
E K  4QTIU                198    0.439  90.323  -0.236   0.069   0.386   0.545
E G  4QTIU                199    0.363  28.584  -2.348   0.020   0.205   0.775
E N  4QTIU                200    0.476  69.760  -1.076   0.020   0.205   0.775
E S  4QTIU                201    0.440  51.521  -0.491   0.019   0.141   0.840
E T  4QTIU                202    0.520  72.110  -0.694   0.056   0.142   0.802
E H  4QTIU                203    0.526  95.625  -0.920   0.052   0.084   0.864
E G  4QTIU                204    0.477  37.548  -0.274   0.052   0.084   0.864
B C  4QTIU                205    0.079  11.148  -2.020   0.052   0.084   0.864
E S  4QTIU                206    0.496  58.108  -0.143   0.018   0.088   0.893
E S  4QTIU                207    0.391  45.790  -1.395   0.056   0.142   0.802
E E  4QTIU                208    0.637 111.371  -0.032   0.056   0.142   0.802
E E  4QTIU                209    0.491  85.743  -0.721   0.052   0.084   0.864
E T  4QTIU                210    0.378  52.373  -1.093   0.019   0.141   0.840
E F  4QTIU                211    0.439  88.188  -0.731   0.022   0.359   0.619
E L  4QTIU                212    0.422  77.305  -0.478   0.022   0.552   0.426
B I  4QTIU                213    0.195  36.001  -0.783   0.023   0.655   0.322
E D  4QTIU                214    0.495  71.373  -0.181   0.023   0.655   0.322
B C  4QTIU                215    0.087  12.229  -1.628   0.021   0.451   0.528
E R  4QTIU                216    0.501 114.752  -0.751   0.020   0.205   0.775
E G  4QTIU                217    0.443  34.888  -1.318   0.018   0.047   0.935
E P  4QTIU                218    0.499  70.780  -1.273   0.018   0.019   0.964
E M  4QTIU                219    0.543 108.694  -1.787   0.018   0.019   0.964
E N  4QTIU                220    0.486  71.165  -1.441   0.018   0.088   0.893
E Q  4QTIU                221    0.296  52.919   0.809   0.021   0.279   0.699
B C  4QTIU                222    0.083  11.667  -1.098   0.023   0.655   0.322
B L  4QTIU                223    0.146  26.678   0.036   0.021   0.756   0.223
E V  4QTIU                224    0.430  66.091  -0.080   0.003   0.718   0.279
B A  4QTIU                225    0.124  13.632  -1.816   0.004   0.616   0.381
E T  4QTIU                226    0.436  60.515  -0.770   0.004   0.616   0.381
B G  4QTIU                227    0.238  18.746  -1.226   0.003   0.718   0.279
E T  4QTIU                228    0.390  54.079  -0.484   0.021   0.756   0.223
E H  4QTIU                229    0.295  53.606  -0.052   0.023   0.655   0.322
E E  4QTIU                230    0.423  73.951  -0.691   0.021   0.451   0.528
E P  4QTIU                231    0.407  57.810  -1.402   0.021   0.279   0.699
E K  4QTIU                232    0.594 122.227  -1.563   0.018   0.088   0.893
E N  4QTIU                233    0.496  72.614  -1.301   0.019   0.141   0.840
E Q  4QTIU                234    0.321  57.420  -0.121   0.022   0.552   0.426
E S  4QTIU                235    0.408  47.782  -1.158   0.004   0.616   0.381
B Y  4QTIU                236    0.164  35.068   0.813   0.023   0.655   0.322
E M  4QTIU                237    0.319  63.832  -0.422   0.023   0.655   0.322
B V  4QTIU                238    0.092  14.125  -0.483   0.022   0.552   0.426
E R  4QTIU                239    0.331  75.845   0.182   0.021   0.451   0.528
B G  4QTIU                240    0.189  14.866  -1.288   0.021   0.451   0.528
B C  4QTIU                241    0.067   9.449  -0.351   0.021   0.451   0.528
B A  4QTIU                242    0.207  22.789  -0.184   0.066   0.296   0.638
E T  4QTIU                243    0.421  58.420   0.302   0.056   0.142   0.802
E A  4QTIU                244    0.485  53.425  -0.797   0.113   0.087   0.800
E S  4QTIU                245    0.478  55.986  -0.589   0.113   0.087   0.800
E M  4QTIU                246    0.334  66.733  -0.332   0.125   0.227   0.648
B C  4QTIU                247    0.057   8.045  -0.046   0.135   0.317   0.548
E Q  4QTIU                248    0.484  86.425  -0.025   0.135   0.317   0.548
E H  4QTIU                249    0.377  68.631  -0.902   0.135   0.317   0.548
E A  4QTIU                250    0.475  52.301  -0.540   0.125   0.227   0.648
E H  4QTIU                251    0.386  70.122  -1.102   0.064   0.216   0.721
B L  4QTIU                252    0.229  41.967  -1.126   0.064   0.216   0.721
B G  4QTIU                253    0.228  17.904  -0.628   0.056   0.142   0.802
E D  4QTIU                254    0.534  76.892  -1.055   0.018   0.088   0.893
E A  4QTIU                255    0.436  48.058  -1.611   0.052   0.084   0.864
B F  4QTIU                256    0.247  49.593  -1.569   0.018   0.088   0.893
E S  4QTIU                257    0.464  54.439  -1.029   0.019   0.141   0.840
E M  4QTIU                258    0.439  87.804  -1.521   0.020   0.205   0.775
E N  4QTIU                259    0.428  62.659  -1.193   0.021   0.279   0.699
E H  4QTIU                260    0.423  76.853  -1.080   0.004   0.514   0.481
B I  4QTIU                261    0.135  25.012  -0.156   0.004   0.616   0.381
E D  4QTIU                262    0.384  55.320  -0.600   0.023   0.655   0.322
B V  4QTIU                263    0.084  12.926  -0.115   0.023   0.655   0.322
B S  4QTIU                264    0.208  24.331  -0.798   0.022   0.552   0.426
B C  4QTIU                265    0.048   6.683  -0.127   0.021   0.451   0.528
B C  4QTIU                266    0.071  10.011  -0.567   0.022   0.359   0.619
E T  4QTIU                267    0.476  65.980  -0.697   0.018   0.088   0.893
E K  4QTIU                268    0.471  96.782  -1.644   0.018   0.019   0.964
E S  4QTIU                269    0.637  74.645  -1.740   0.018   0.019   0.964
E G  4QTIU                270    0.421  33.133  -0.941   0.018   0.019   0.964
B C  4QTIU                271    0.083  11.723  -1.410   0.018   0.019   0.964
E N  4QTIU                272    0.360  52.748  -1.178   0.018   0.019   0.964
E H  4QTIU                273    0.402  73.197  -0.394   0.018   0.019   0.964
E P  4QTIU                274    0.506  71.816  -0.608   0.058   0.017   0.925
E D  4QTIU                275    0.307  44.296  -1.348   0.058   0.017   0.925
E L  4QTIU                276    0.418  76.499  -0.585   0.115   0.016   0.868
E D  4QTIU                277    0.550  79.197  -0.533   0.115   0.016   0.868
E V  4QTIU                278    0.443  68.043   0.744   0.113   0.043   0.844
E Q  4QTIU                279    0.518  92.533  -0.398   0.113   0.087   0.800
E Y  4QTIU                280    0.378  80.714  -0.710   0.113   0.087   0.800
E R  4QTIU                281    0.412  94.371  -1.636   0.113   0.087   0.800
E S  4QTIU                282    0.548  64.190  -1.243   0.053   0.043   0.903
E G  4QTIU                283    0.902  70.956  -2.285   0.003   0.003   0.994

Explain the output.  Go back.
```

---
